# The timeliness of COVID-19 testing and tracing in eight public health regions in the Netherlands: Jizzo Bosdriesz

**DOI:** 10.1093/eurpub/ckac130.020

**Published:** 2022-10-25

**Authors:** JR Bosdriesz, EM den Boogert, NHTM Dukers-Muijrers, HM Götz, IE Goverse, T Leenstra, SFH Raven, SKS van Dijken, K Wevers, AA Matser

**Affiliations:** 1 Department of Infectious Diseases, Public Health Service of Amsterdam, Amsterdam, Netherlands; 2 Department of Internal Medicine, Division of Infectious Diseases, Amsterdam UMC, Amsterdam, Netherlands; 3 Department of Infectious Disease Control, Municipal Health Service Hart voor Brabant, ‘s-Hertogenbosch, Netherlands; 4 Centre for Infectious Disease Control, RIVM, Bilthoven, Netherlands; 5 Department of Sexual Health and Infectious Diseases, Public Health Service South Limburg, Heerlen, Netherlands; 6 Department of Health Promotion, Maastricht University, Maastricht, Netherlands; 7 Department of Infectious Disease Control, Municipal Public Health Service Rotterdam-Rijnmond, Rotterdam, Netherlands; 8 Department of Public Health, Erasmus MC, University Medical Center, Rotterdam, Netherlands; 9 Department of Infectious Diseases, Municipal Health Service Groningen, Groningen, Netherlands; 10 Department of Infectious Diseases, Public Health Service region Utrecht, Utrecht, Netherlands; 11 Department of Infectious Diseases, Public Health Service Flevoland, Lelystad, Netherlands; 12 Department of Infectious Disease Control, Municipal Health Services Gelderland Midden, Arnhem, Netherlands

## Abstract

**Background:**

Testing and Contact Tracing (TCT) was a core strategy in the fight against the spread of SARS-CoV-2. However, little is known about the real-world effectiveness of TCT for COVID-19. Because time is an important conditional factor, we aim to study timeliness of TCT in the Netherlands, and its determinants.

**Methods:**

We used routine COVID-19 TCT registry data from all individuals who tested positive for SARS-CoV-2 at 8 Dutch regional public health services from 1-6-2020 to 28-2-2021 (N = 338,066). We calculated median time intervals of TCT stages. Factors associated with the time between test result and completion of TCT, categorised as ≤ 3 days and >3 days, were assessed using logistic regression adjusting for region, testing site, and laboratory. Potential determinants were: gender, age, country of birth, number of close contacts, working in health-care or education, TCT manpower, and the Oxford Covid-19 Government Response Tracker (OxCGRT).

**Results:**

The median time from symptom onset to TCT completion was 6 days (IQR:3-10). Median times between TCT stages were 1 day (IQR:0-3) for symptom onset to test request, 1 day, (IQR:0-1) for test request to sample collection, 1 day, (IQR:1-1) for sample collection to test result, and 2 days (IQR:1-5) for test result to TCT completion. In 31.7% of tests, time between test result and TCT completion was >3 days. This delay was associated with being older (65+), whereas being younger (0-14), a higher OxCGRT, scaling down TCT, and a higher number of TCT employees were associated with a shorter interval.

**Conclusions:**

Over fifty percent of interval times from symptom onset to TCT completion exceeded the median SARS-CoV-2 incubation period of 5 days. There seems to be little room for improvement on the side of the index case, but there are some implications for logistics such as increasing TCT manpower, and better integration of digital systems.

**Key messages:**

- Testing and contact tracing were key elements of fighting the spread of COVID-19, but the potential impact was reduced because >50% of interval times exceed the median SARS-CoV-2 incubation period.
- The influence of contextual factors shows that stricter government policies, and increasing TCT manpower could help speed up the process of TCT for COVID-19.

